# Complement’s C1 Complex, Factor H and the X Factor: A Personal Tribute to Prof. Robert B. Sim

**DOI:** 10.3390/v13050793

**Published:** 2021-04-29

**Authors:** Uday Kishore

**Affiliations:** Biosciences, College of Health, Medicine and Life Sciences, Brunel University London, Uxbridge UB8 3PH, UK; uday.kishore@brunel.ac.uk or ukishore@hotmail.com

It is with great sadness that I am writing this obituary for my mentor, colleague and friend, Bob, i.e., Prof. Robert B. Sim, who passed away on 6 February 2021. Bob was battling cancer for over 8 months. Tragically, fate took this great complementologist away from us too early. Bob was a legend in the field of complement; his passing is a big loss to the field of innate immunity and will be felt more profoundly by his numerous students, post-doctoral scientists, principal investigators and close friends whom he encouraged, supported and looked after.

Bob was born in Crieff, Scotland, and carried out his secondary schooling at the Perth Academy, Scotland (1963–1968), where he studied physics, chemistry, mathematics and biology in addition to Latin and French. Between 1969 and 1973, he studied for a BSc Honors degree at the University of Edinburgh in Biochemistry (Figure 1) and was awarded a Distinction via five first class merit certificates, two medals and the Boots Drummond Prize. Between 1973 and 1976, Bob carried out his DPhil work at the University of Oxford (Wolfson College) in the Biochemistry Department. His research thesis was titled “The First Component of Human Complement” and was conducted within the Medical Research Council (MRC) Immunochemistry Unit, Oxford, under the supervision of the Nobel Prize winner Prof. Rodney Porter and Prof. Ken Reid, FRS. Bob also had the distinction of being a Graduate Scholar of Wolfson College, Oxford.

In 1976, Bob moved to France and was the holder of a French Exchange post-doctoral Fellowship arranged by the Medical Research Council (UK) and INSERM (France). He worked within the Department of Fundamental Research (Biochemistry) at the Centre d’Etudes Nucleaires, Grenoble, France, in a group led by Professor M.G. Colomb (Figure 2). After his return to the UK in 1978, Bob started working at the MRC Immunochemistry Unit at Oxford University as a member of the scientific staff, and later, he was also appointed as University Research Lecturer, which continued until 2008, when he retired as Deputy Head of the MRC Unit. His retirement did not hold him back, as he remained very engaged with research via his honorary association with the University of Oxford (Departments of Biochemistry and Pharmacology), the University of Leicester (Department of Infection, Immunity and Inflammation), Brunel University (Centre for Infection, Immunity and Disease Mechanisms) and Kingston University (Faculty of Science and Engineering). In 2017, Bob received an Honorary Doctorate from the University of the Republic of Uruguay, Montevideo.

Bob was a prolific scientist (Robert B Sim–Google Scholar, https://scholar.google.co.uk/citations?user=eOGvDm4AAAAJ&hl=en&oi=ao, accessed on 1 April 2021). His pioneering research included the identification of the activation mechanism of C3, association and activation of the C1 complex and MASPs; purification and characterization of several effectors and regulators of the alternative and lectin pathways, C1q/collectin receptor, IgG glycosylation in human health and disease, complement–pathogen interaction, regulation of complement and collectins in inflammatory diseases, complement–nanoparticle interaction and complement’s link with coagulation (Sim RB–Search Results–PubMed (https://pubmed.ncbi.nlm.nih.gov/?term=sim+RB&sort=pubdate, accessed on 1 April 2021)). However, these areas are mere snippets; his versatility went beyond the realm of complement (Robert B Sim–researchgate.net (https://www.researchgate.net/profile/Robert-Sim-2, accessed on 1 April 2021)). In 2013, his outstanding contributions to the complement research field were recognized by a lifetime achievement award from the European Complement Network (Figure 3).

I got to work and collaborate closely with Bob after I had moved from the MRC Immunochemistry Unit (where I worked with Prof Ken Reid for two years) to the Weatherall Institute of Molecular Medicine, John Radcliffe Hospital, Oxford. Bob and I examined a potential role of factor H as a regulator of C1q for certain ligands. This research led to another study that assessed factor H interaction with apoptotic and necrotic cells. I later moved to Brunel University London, which coincided with the closure of the MRC Immunochemistry Unit in 2008 when Ken (Prof. Reid) retired. Bob and I continued our collaboration on the roles of factor H and properdin in microbial infection. In the last 5 years, until Bob became very ill, we continued working and publishing together in the area of complement and nanoparticles. We also studied other functions of complement proteins, including factor H, C4BP and properdin (Sim RB and kishore U—Search Results—PubMed (https://pubmed.ncbi.nlm.nih.gov/?term=sim%20RB%20and%20kishore%20u&sort=pubdate, accessed on 1 April 2021)).

Consequently, over a couple of decades, I came to know the two sides of Bob: a passionate and meticulous scientist, but also a charismatic warm-hearted friend and devoted family man. When it came to his science, he had no time for exchanging pleasantries. Young researchers, especially PhD students, were fully aware that they needed to carry out experiments rigorously before or after a planned discussion with Bob. It was common to see phrases such as “rubbish”, “crap”, “if I do not get it, who else will”, “No, no, no-you cannot say that” in a first draft of a manuscript when Bob returned it to me with his feedback. His comments were often given in “track changes” and were red, capitalized, bold and underlined! His “You cannot miss them” approach to edits was Bob’s idea of utilizing the creative potential of the “track changes” function!

Bob was an extraordinarily generous mentor to me (Figure 4)**.** He must have made several dozen trips to our laboratory, bringing with him endless reagents (especially those needing a home after the MRC Unit closed), and with each reagent, there was/is a hand-written (96 font size) detailed note: buffer, history, previous results, etc. Bob was also a great story teller—about science, his past experience with projects and people. He loved to spend time with PhD students, often having insightful discussions on a range of topics. My team members adored him as much as they were worried about showing him their first draft of a manuscript. In various moving tributes during his funeral in March 2021, it was obvious how well Bob mentored his former students (supervising over 30 PhD students), many of whom continued to collaborate with him, long after they left Oxford.

Despite his almost uncompromising streak when it came to research integrity and quality, Bob was very friendly, kind-hearted and affectionate and had a great sense of humor. He was incredibly knowledgeable but modest, self-critical but pragmatic, and a thorough scientist but a compassionate teacher. Bob never sought external recognition and, hence, was always his own man. He always kept himself free from the need of professional networking for anything other than doing research.

I feel very privileged that I worked with him and shared ideas as well as jokes and referees’ comments over numerous lunches/drinks in Oxford and London. Even a few days before his passing, we talked about finishing a couple of outstanding papers. He kept his sense of humor and wit alive even in his last week, telling me how he uses his laptop these days as if he were learning to play piano!

As we say: “there is no goodbye for friends; they will always live in our heart!”

## Figures and Tables

**Figure 1:**
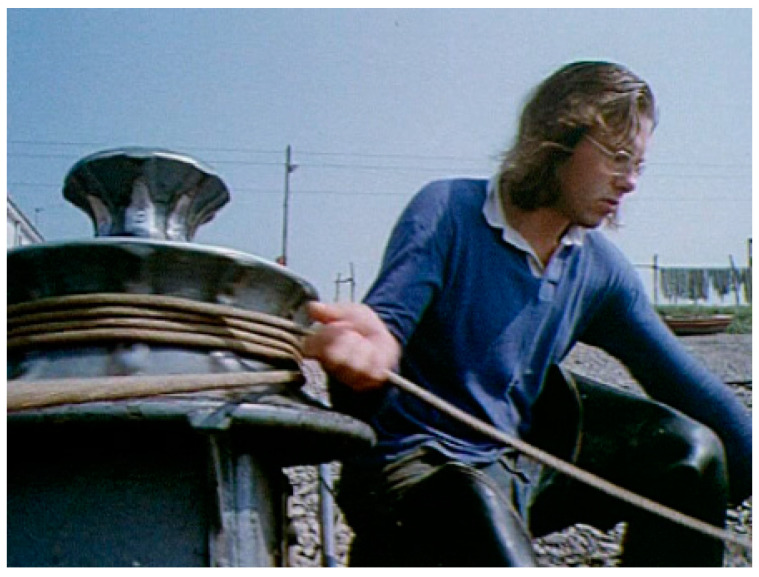
Bob spent his summers and also some Easter vacations working as a net fisherman for the Tay Salmon Fisheries Company between 1968 and 1974. He is seen here in his waders sitting by the winch, which pulled in the net at the fishing station Venture on the north bank of the Tay, in a still picture taken from a promotional film to promote the region. He was a gaffer or foreman of his crew from 1970 onwards.

**Figure 2:**
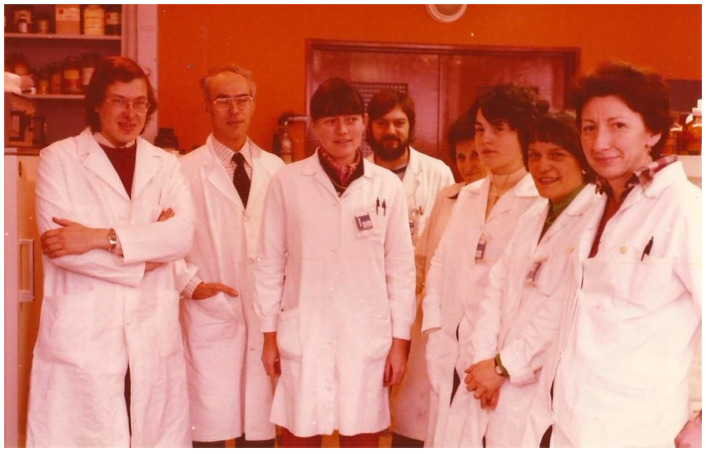
From 1976 to 1978, Bob worked mainly on the C1 complex and C1-inhibitor in the Centre d’Etudes Nucleaires in Grenoble in the laboratory of Maurice Colomb (white coats were compulsory). This work was important in Bob’s subsequent interaction with John Jackson from Dublin in determining the cause of autoimmune angioedema. From left to right are Bob Sim, Maurice Colomb, Angeline Reboul, Gerard Arlaud, Renee Ducret, Marie Bernadette Villiers, Mme Dodu and Mme Duplas.

**Figure 3:**
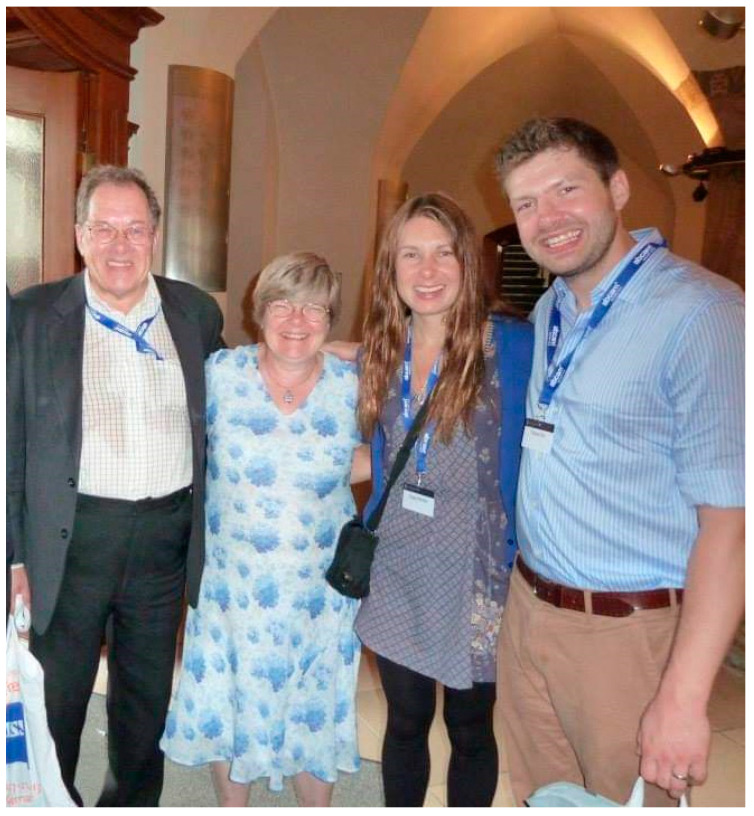
Bob’s outstanding contributions to the complement research field were recognized in 2013, when he received a lifetime achievement award from the European Complement Network (ECN). Bob was a devoted family man. He leaves behind Edith (his wife of over 45 years), two children (Francis and Grace), and four grandchildren (Charles, Stanley and Wilfred Sim, and Mary Mackintosh). From left: Bob, Edith, Grace and Francis at Jena, Germany, after the ECN award ceremony.

**Figure 4:**
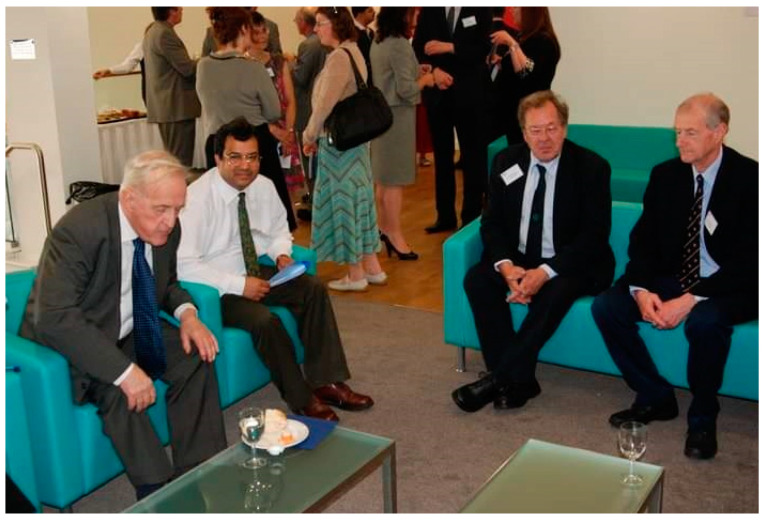
Bob attending the opening ceremony of the Centre for Infection, Immunity and Disease Mechanisms at the Brunel University London in 2009. From the left: Sir David Weatherall, Uday Kishore, Bob, and Prof Ken Reid.

